# Assessing malaria transmission in a low endemicity area of north-western Peru

**DOI:** 10.1186/1475-2875-12-339

**Published:** 2013-09-22

**Authors:** Angel Rosas-Aguirre, Alejandro Llanos-Cuentas, Niko Speybroeck, Jackie Cook, Juan Contreras-Mancilla, Veronica Soto, Dionicia Gamboa, Edwar Pozo, Oscar J Ponce, Mayne O Pereira, Irene S Soares, Michael Theisen, Umberto D’Alessandro, Annette Erhart

**Affiliations:** 1 Instituto de Medicina Tropical, Alexander von Humboldt, Universidad Peruana Cayetano Heredia, Lima, Peru; 2 Research Institute of Health and Society (IRSS), Université catholique de Louvain, Brussels 1200, Belgium; 3 Department of Medicine Solna, Karolinska Institutet, Malaria Research Unit, Stockholm, Sweden; 4 Departamento de Ciencias Celulares y Moleculares, Facultad de Ciencias y Filosofia, Universidad Peruana Cayetano Heredia, Lima, Peru; 5 Sub-region de Salud Luciano Castillo Coloma, Sullana, Peru; 6 Departamento de Análises Clínicas e Toxicológicas, Faculdade de Ciências Farmacêuticas, Universidade de São Paulo, São Paulo, Brazil; 7 Department of Clinical Biochemistry and Immunology, Statens Serum Institut, Copenhagen, Denmark; 8 Department of Infectious Diseases, Copenhagen University Hospital and Centre for Medical Parasitology at Department of International Health, Immunology, and Microbiology, University of Copenhagen, Rigshospitalet, Copenhagen, Denmark; 9 Disease Control and Elimination, Medical Research Council Unit, Fajara, The Gambia; 10 Institute of Tropical Medicine, Antwerp 2000, Belgium

**Keywords:** Malaria transmission intensity, Low endemicity, Elimination, Polymerase chain reaction, Serology, Peru

## Abstract

**Background:**

Where malaria endemicity is low, control programmes need increasingly sensitive tools for monitoring malaria transmission intensity (MTI) and to better define health priorities. A cross-sectional survey was conducted in a low endemicity area of the Peruvian north-western coast to assess the MTI using both molecular and serological tools.

**Methods:**

Epidemiological, parasitological and serological data were collected from 2,667 individuals in three settlements of Bellavista district, in May 2010. Parasite infection was detected using microscopy and polymerase chain reaction (PCR). Antibodies to *Plasmodium vivax* merozoite surface protein-1_19_ (PvMSP1_19_) and to *Plasmodium falciparum* glutamate-rich protein (PfGLURP) were detected by ELISA. Risk factors for exposure to malaria (seropositivity) were assessed by multivariate survey logistic regression models. Age-specific antibody prevalence of both *P. falciparum* and *P. vivax* were analysed using a previously published catalytic conversion model based on maximum likelihood for generating seroconversion rates (SCR).

**Results:**

The overall parasite prevalence by microscopy and PCR were extremely low: 0.3 and 0.9%, respectively for *P. vivax,* and 0 and 0.04%, respectively for *P. falciparum,* while seroprevalence was much higher, 13.6% for *P. vivax* and 9.8% for *P. falciparum*. Settlement, age and occupation as moto-taxi driver during previous year were significantly associated with *P. falciparum* exposure, while age and distance to the water drain were associated with *P. vivax* exposure. Likelihood ratio tests supported age seroprevalence curves with two SCR for both *P. vivax* and *P. falciparum* indicating significant changes in the MTI over time. The SCR for PfGLURP was 19-fold lower after 2002 as compared to before (λ1 = 0.022 *versus* λ2 = 0.431), and the SCR for PvMSP1_19_ was four-fold higher after 2006 as compared to before (λ1 = 0.024 *versus* λ2 = 0.006).

**Conclusion:**

Combining molecular and serological tools considerably enhanced the capacity of detecting current and past exposure to malaria infections and related risks factors in this very low endemicity area. This allowed for an improved characterization of the current human reservoir of infections, largely hidden and heterogeneous, as well as providing insights into recent changes in species specific MTIs. This approach will be of key importance for evaluating and monitoring future malaria elimination strategies.

## Background

Despite recent reduction in its estimated incidence, malaria remains the most important human arthropod-borne disease, worldwide and in the Americas [1]. In Peru, malaria incidence has fluctuated dramatically over the last 50 years. Between 1954 and 1967, malaria was well under control by coordinated eradication efforts (resulting in only 1,500 cases in 1965) [2], but returned to high levels when eradication campaigns ceased. Following the occurrence of drug-resistant *Plasmodium falciparum* strains to chloroquine (CQ) and sulphadoxine-pyrimethamine (SP) [3,4] and the spread of *Anopheles darlingi* in the Amazon Region [5], malaria re-emerged in the 1990s, reaching a peak of more than 200,000 cases in 1998 after the El Niño Southern Oscillation (ENSO) climatologic phenomenon [6]. Between 2000 and 2005, the annual incidence fluctuated between 70,000 and 80,000 cases followed by a steady decrease until 2011, when 22,877 cases were reported [7]. This achievement can be attributed to the strengthening of the National Malaria Control Programme (NMCP) and to the implementation of comprehensive interventions such as the use of artemisinin-based combination therapy (ACT), the distribution of long-lasting insecticidal mosquito nets (LLINs), and health education campaigns with the strong support of international donors, e g, US Agency for International Development (USAID), and the Global Fund for Aids, Tuberculosis and Malaria (GFATM) [8-10].

In Peru, the significant decline in malaria incidence over the past decade has modified its epidemiological profile and consequently calls for adapted control strategies. Currently, there is the need for targeting interventions and surveillance to foci of residual transmission in the north-west coast as well as in the Peruvian Amazon region. A key element for this re-orientation will be the availability of accurate measurement of malaria transmission intensity (MTI) and its evolution in space and time [11,12]. However, it is not clear how best to monitor changes in transmission and disease burden in low endemicity areas [13].

Traditional methods to measure MTI include the collection of entomological and parasitological parameters. However, in areas of low endemicity, these measures are often subject to fluctuations for reasons other than true changes in transmission. For example, the improvement of case detection methods usually leads to an artificial increase in malaria incidence, which makes difficult the comparison between pre- and post-intervention data. Furthermore, entomological (entomological inoculation rate (EIR)) and parasitological measures (infections detected by microscopy) estimated through community surveys require very large sample sizes and are therefore extremely time and money consuming, while not able to catch seasonal variations [14,15]. Moreover, parasitological surveys using microscopy cannot detect subpatent infections which are commonly reported in areas of low transmission [16,17]. Molecular tests such as polymerase chain reaction (PCR) are much more sensitive for the detection of low parasite-density infections, and will become increasingly important for malaria elimination programmes [18,19].

Serological markers have recently been used in several endemic areas across the world to estimate MTI [20-24] and monitor its changes over time following interventions [25]. Serological techniques are particularly suited to low endemic areas as antibodies remain in the blood longer than malaria parasites and are thus easier to detect and less subject to seasonal variations. This paper reports on population-based estimates of *P. falciparum* and *Plasmodium vivax* prevalence and seroprevalence in an area of low endemicity in the north-west coast of Peru. The combination of molecular and serological tools is aimed at improving the detection of current malaria infections as well as recent exposure, in order to get insights into the residual transmission dynamics.

## Methods

### Study area

**Figure 1:**
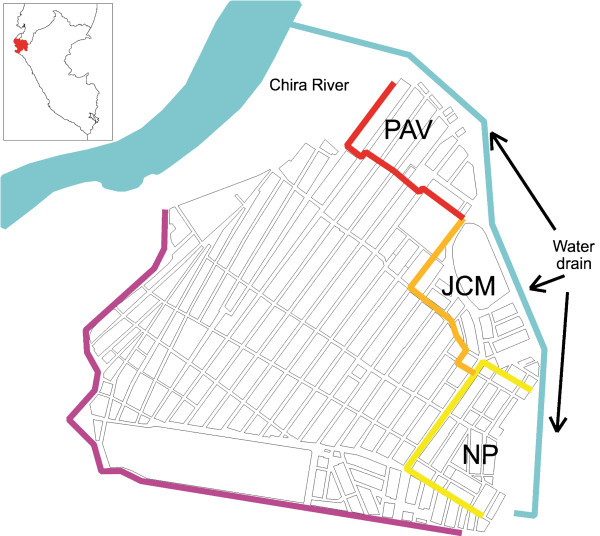
**Study area in Bellavista district (Piura department, northwestern coast of Peru) including three settlements.** Pavletich (PAV), Jose Carlos Mariátegui (JCM) and Nuevo Porvenir (NP).

The study was conducted in Bellavista, a small district of Sullana province located in Piura department, north-western coast of Peru (Figure 1). Piura is historically considered an important focus of malaria transmission in Peru [26]. Bellavista district is a peri-urban settlement (37,000 inhabitants in 2010) directly connected to Sullana city, at 30 km from Piura, the fifth largest city in Peru. Three contiguous squatter settlements were selected for the study: Pavletich (PAV, nine blocks of houses), Jose Carlos Mariátegui (JCM, 12 blocks) and Nuevo Porvenir (NP, 13 blocks) based on their malaria endemicity (between 2008 and 2010 most of the district malaria cases occurred in these settlements) and their proximity to the largest water drain called *Boqueron* (=big hole). Situated at the eastern edge of the settlements, this artificially created drain flows from south to north for about 2 km (maximum width 100 m, depth 30 m) to end into the Chira River.

The winter season is from May to November and the hot and rainy summer from December to April. This seasonal pattern is periodically altered by the ENSO phenomenon [27] with torrential rain and strong winds that can cause flooding and landslides. In 2010, annual rainfall, average relative humidity and average temperature were 103 mm, 76% and 24.2°C, respectively [28]. The main vector is *Anopheles albimanus* and the vast majority of malaria cases are due to *P. vivax*[29]. The main occupations in the area are informal trade, agriculture and small animal farming.

### Data collection

A full census of the study population was carried out in April 2010, collecting individual data on sociodemographic (age, gender, education, economic activities, income) as well as on malaria prevention characteristics. Each house was identified with a unique code number and georeferenced using a handheld global positioning system (GPS) device (Garmin’s GPSMAP 60CSx) that also calculated the closest distance to the water drain. Each individual was given a seven-digit unique code number combining the village, household and individual code.

The cross-sectional survey was carried out in May 2010 and included all residents in the study area. Each household was visited, and a written informed consent was sought from each participant. If part of, or the whole household, was absent at the time of survey, the study team would return within the next two days to maximize subject participation. Each participant was examined for fever and malaria symptoms, and a finger-prick blood sample was taken for immediate microscopy (thick and thin blood smears) and later serological and molecular tests at Institute of Tropical Medicine Alexander von Humboltd, Lima (ITM-AvH). Filter paper (Whatman grade 3, Whatman, Springfield Mill, USA) dried blood samples were individually stored at 4°C with desiccant until processed at the ITM-AvH. Infected individuals (presence of *Plasmodium* trophozoites) were treated according to the national guidelines.

Additionally, retrospective data on annual malaria incidence by species from 1990 to 2010, registered by the Office of Epidemiology at Regional Health Direction of Piura, were obtained from the National Institute of Statistics and Informatics [7,28]. In Peru, weekly notification of confirmed malaria cases (by standard microscopy) at health facilities and data aggregation at district and at departmental level are mandatory. Patients presenting with symptoms compatible with malaria at health facilities are systematically tested by microscopy and treated following national guidelines.

### Laboratory procedures

#### Microscopy

Thick and thin smears were stained for 10 min with a 10% Giemsa solution, and parasite density was expressed as the number of parasites/μl, after counting a total of 200 white blood cells (WBC) (or 500 WBCs if less than ten parasites/field), and assuming an average of 6,000 WBCs/μl according to the national guidelines [30]. Microscopy examination was performed immediately after sample collection at the Reference Laboratory in Sullana, and later quality control was done blindly on all positive slides and 10% of randomly chosen negative slides by a senior technician at ITM-AvH. Any discordant results were reread by a second senior technician until agreement.

#### Species-specific polymerase chain reaction (ss-PCR)

Parasite DNA was extracted using the saponin Chelex 100 method [31]. Briefly, filter-paper blood spots containing approximately 20 μl of blood were cut into pieces of approximately 5 sq mm, and incubated with 20 μl of 0.05% saponin at room temperature for four hours. Then, 10 μl of 20% Chelex 100 solution was added, and the sample was incubated for 10 min at 95°C, followed by a centrifugation at 11,000 g. The supernatant (DNA) was transferred into a new tube and stored at −20°C until PCR was performed. The DNA was amplified by a semi-nested multiplex PCR method targeting the 18S rDNA region, as described by Rubio *et al*[32]. PCR products were analysed in a 2% agarose gel with a standard 100 bp DNA lader, using ethidium bromide staining (0.5 μg/ml) and a data image Analyzer with UV trans-illuminator.

#### Serology

*Plasmodium falciparum* glutamate-rich protein (PfGLURP) and *P. vivax* merozoite surface protein1_19_ (PvMSP1_19_) antibodies were detected using an ELISA protocol published elsewhere [33]. Briefly, dried blood filter-paper samples (5 mm diameter disc/sample) were eluted overnight at 4°C in 2 ml of PBS-Blotto-Tween. Two hundred μl of the eluate were added in duplicate to blocked ELISA plates (Plate Chamaleon, Hydex) coated separately with *P. falciparum* GLURP R2 [34] and *P. vivax* MSP1-_19_[35]. Pooled sera from five *P. falciparum-* or *P. vivax*-infected patients, and from five non-infected control group were diluted at 1:400 in PBS-Blotto, as positive and negative control, respectively. Goat anti-human IgG (H + L) peroxidase (Sigma, affinity purified) diluted to 1: 20,000 in PBS-Tween was used as conjugate and incubated for one hour before development of the ELISA using 200 μl ABTS substrate-chromogen solution. Optical densities (ODs) were read at 405 nm, and corrected OD values were computed by subtracting the mean OD of the antigen negative control wells from the mean OD of the corresponding antigen containing wells. To ensure a standardization of the sample results across ELISA plates, the percent positivity (PP) of each specimen was calculated using the OD of the positive control serum as 100%. Quality control was done blindly at ITM-AvH on 5% of randomly chosen samples. The cut-off for positivity was generated using a mixture model as previously described by Corran *et al*. [36].

#### Data management and statistical analysis

Data were double entered, validated and cleaned in Excel (Microsoft Corp, USA), and data analysis was performed with Stata v.11 (Stata Corp, College Station, USA) and R v.2.15 software (R Development Core Team, R Foundation for Statistical Computing, Austria).

Malaria infection by PCR was defined as an individual with a positive PCR result, regardless of symptoms. Clinical malaria was defined as a patient with fever (body temperature >37.5°C), and/or history of fever in the previous two days, and positive for malaria by microscopy and/or PCR. As maternally derived antibodies can be found in young infants [37], malaria exposure was defined as an individual older than six months with a positive ELISA test for either or both *P. falciparum* and *P. vivax*. Descriptive statistics were used to calculate malariometric indices (parasite and antibody prevalence). Uni- and multivariate adjusted analyses were performed using survey logistic regression to determine risk factors for malaria infection (PCR positive) or malaria exposure (serology positive) adjusting for all potential confounders such as: settlement, gender, age, income, education, occupation during previous year, bed net use, distance to the water drain, and movements outside the settlement during previous year. A p-value <0.05 was considered significant for risk factors to be included in the multivariate adjusted model. Interactions were systematically checked for up to order two. All analyses took into account the survey design characteristics, using settlement as strata and household as primary sampling unit.

A simple reversible catalytic conversion model was used to fit the dichotomized serological results, using maximum likelihood methods as published by Drakeley *et al*. [22]. Briefly, this model generates age-seroprevalence curves, allowing for the estimation of the force of infection, or seroconversion rate (SCR, λ), and a seroreversion rate (ρ). Upon visual examination, if age-seroprevalence data suggest an obvious step at a certain age (time point), a model allowing for two forces of infection (λ_1_ and λ_2_) and related profile likelihood plots are run to determine the most likely time point for change in SCR, as previously described by Stewart *et al*. [24]. The identified time point is subsequently incorporated into the catalytic model to fit a seroprevalence curve with two forces of infection (before and after time cut-off) [38], which is then compared -using the likelihood ratio (LR) test (p < 0.05)- with a sero-prevalence curve with only one force of infection.

### Ethical issues

Permission was received from health and local authorities after explaining the purpose and procedures of the survey. Signed, informed consent was obtained prior to participation by all adults and the parents of all participating children <18 years. In addition to their parent’s consent, children ≥ seven years old provided signed informed assent prior to participating. Ethical clearance was obtained from the Ethics Review Board of the Universidad Peruana Cayetano Heredia, Lima, Peru (SIDISI code: 056736).

## Results

A total of 996 households and 4,650 individuals were identified during the census, and were distributed over the three study settlements as follows: PAV (433 houses; 2,163 individuals), JCM (312; 1,358) and NP (251; 1,129). The overall sex ratio was 0.94, and the age distribution showed that half of the population was <20 years old. A total 2,733 individuals (58.8%) living in 871 households (87.4%) were available at the time of the survey and 97.6% (2,667/2,733) of them accepted to participate. Females (61%) slightly outnumbered males, but the age distribution was very similar to the one in the total population. Among adults aged ≥18 years old, 60% had completed secondary education level. Only about 20% of the respondents had a regular income, almost all below the minimum national vital wage. The most commonly reported occupations were housewife (27.1%), labourer (9.0%) and trader (6.4%), while preprofessionals (children, students) and retired represented half of the participants. Very few respondents reported having been working as farmer (2.4%) or moto-taxi driver (2.6%) in the past year. More than half (52.6%) of the participants stated they were regularly sleeping under an untreated net, and 11.5% had experienced at least one confirmed *P. vivax* episode during the previous year (Table 1).

**Table 1:** Baseline characteristics of the study population (N = 2,667)

		**n**	**%**
**Settlement**			
	Pavletich (PV)	1,128	42.3
	José Carlos Mariategui (JCM)	797	29.9
	Nuevo Porvenir (NP)	742	27.8
**House distance to water drain (m)**			
	<100	183	6.9
	100–250	1401	52.5
	>250	1,083	40.6
**Gender**			
	Female	1,628	61.0
	Male	1,039	39.0
**Age groups (years)**			
	0–5	447	16.8
	6–10	404	15.1
	11–19	491	18.4
	20–39	889	33.3
	> = 40	436	16.4
**Education (> = 18 years, n = 1,421)**			
	None	44	3.1
	Primary	517	36.4
	Secondary	689	48.5
	Superior	171	12.0
Income amount*			
	None	2,081	78.0
	< 650 PEN	458	17.2
	> = 650 PEN	83	3.1
	Missing	45	1.7
**Current economic activity**			
	None (children, students, retired)	1,319	49.5
	Farmer	41	1.5
	Moto-taxi driver	74	2.8
	Housewife	723	27.1
	Trader	172	6.4
	Laborer (indoor, manual worker)	240	9.0
	Others	98	3.7
**Occupation as farmer (previous 12 months)**			
	No	2,604	97.6
	Yes	63	2.4
**Occupation as moto-taxi driver (previous 12 months)**			
	No	2,598	97.4
	Yes	69	2.6
**Bed net use**			
	Never/Sometimes	1,250	46.9
	Always	1,402	52.6
	Missing	15	0.5
**Confirmed *P. vivax *malaria (previous 12 months)**			
	No	2,360	88.5
	Yes (1 episode)	273	10.2
	Yes (> = 2 episodes)	34	1.3

*Income expressed in Peruvian nuevos soles (PEN). The official vital minimum wage in Peru is 650 PEN (approx 230 USD).

Parasite prevalence determined by microscopy was 0.3% (total eight *P. vivax* infections) while by PCR it was estimated at 1% including one *P. falciparum* and 25 *P. vivax* infections (Table 2). Malaria seroprevalence for *P. vivax* and *P. falciparum* was estimated at 13.6 and 9.8%*,* respectively, with 9.0% of the total seropositive individuals having antibodies against both antigens. A very weak correlation between antibody responses (percent positivity = PP) to the two species were found in individuals older than ten years (r = 0.1, p < 0.001), but not in younger children (p > 0.1). Most of the *P. vivax* PCR-positive cases were submicroscopic (17/25 = 68.0%) and asymptomatic (22/25 = 88.0%) at the time of the survey (Table 3). PCR positivity to *P. vivax* was significantly associated with the presence of *P. vivax* parasites (by microscopy) (p < 0.001) or antibodies (p < 0.001), as well as with symptoms (p < 0.001). Association between PCR and sero-positivity to *P. falciparum* could not be explored since only one *P. falciparum* infection was detected by PCR. This infection was asymptomatic and submicroscopic, and was found in a 16-year-old girl who also had antibodies against *P. falciparum*.

**Table 2:** Malaria prevalence (by microscopy and PCR) and seroprevalence

		**n**	**N**	**%**	**95% CI**
**Prevalence by microscopy**	*P. vivax*	8	2,667	0.3	[0.2; 0.6]
Parasite density/μl, median [range]	432 [112; 1,529]				
**Prevalence by PCR**	*P. vivax*	25	2,667	0.9	[0.7; 1.4]
	*P. falciparum*	1	2,667	0.04	[0.01; 0.3]
	Any species	26	2,667	1	[0.6; 1.4]
**Seroprevalence**	PvMSP1_19_	360	2,653	13.6	[12.2; 15.1]
	PfGLURP	259	2,653	9.8	[8.6; 11.1]
	Total	567	2,653	21.4	[19.7; 23.1]

**Table 3:** **Association between *Plasmodium vivax *PCR results and microscopy, serology and symptoms**

		***P. vivax *PCR Negative**		***P. vivax *PCR Positive**		
		**(N = 2642)**		**(N = 25)**		
		**n**	**%**	**n**	**%**	**p-value**
***P. vivax microscopy***						
	Negative	2,642	100	17	68.0	<0.001
	Positive	0	0	8	32.0	
***P. vivax *MSP**_**1-19 **_**serology**						
	Negative	2,282	86.8	11	44.0	<0.001
	Positive	346	13.2	14	56.0	
***P. falciparum *GLURP serology**						
	Negative	2,369	90.2	22	88.0	0.71
	Positive	256	9.8	3	12.0	
**Fever and/or history of fever (previous 2 days)**						
	No	2,592	98.1	22	88.0	<0.001
	Yes	50	1.9	3	12.0	

Given the small number of PCR-positive individuals, a multivariate adjusted risk factor analysis was not carried out, and the univariate analysis showed that distance to water drain was significantly associated with the risk of *P. vivax* infection (no association could be found with age, gender, income, education, bed net, or previous travelling outside the community). Individuals living at <100 m from the water drain had increased odds of *P. vivax* infection than those living farther (≥100 m) (OR = 3.4, 95% CI [1.3–9.0]).

Distance to water drain, age, education, income, occupation as farmer in the previous year, and history of *P. vivax* malaria in the previous year were significantly associated to *P. vivax* exposure in the univariate analysis (Table 4). The multivariate analysis (excluding history of malaria in the previous year) showed that only age and distance to water drain remained independently associated with *P. vivax* seropositivity. Individuals older than ten years had increasingly higher odds of *P. vivax* seropositivity compared to younger children (test for trend p < 0.001), while those living at closer distance to the water drain had increasingly higher odds of exposure to *P. vivax* compared to those living farther than 250 m. A significant interaction (OR = 5.7; p = 0.014) between age groups > ten years and distance <100 m was found, showing that the effect of age (>ten *versus* ≤ ten years) was significantly higher in those living closer (<100 m) to the drain (OR = 11.7; 95 CI [3.0; 45.1]; p < 0.001) compared to those living farther (OR = 2.0; 95 CI [1.5; 2.7], p < 0.001). Conversely, the effect of distance to water (<100 m *versus* ≥100 m) was significant only in the older age group (OR = 2.4, 95 CI [1.6; 3.7], p < 0.001).

**Table 4:** **Multivariate adjusted analysis for the risk of exposure to *Plasmodium vivax *using survey logistic regression**

**Risk factors**		**Positive PvMSP1-19**				**Unadjusted**		**Adjusted ≈**	
		**%**	**95% CI**	**n**	**N**	**OR**	**95% CI**	**OR**	**95% CI**
**Settlement**									
	PAV	12.7	[10.8; 14.9]	143	1,123	1.0		-	
	JCM	15.6	[13.1; 18.5]	123	789	1.3	[0.95; 1.7]	-	
	NP	12.7	[10.0; 15.9]	94	741	1.0	[0.7; 1.4]	-	
**House distance to water drain (m)**									
	>250	9.5	[7.8; 11.6]	103	1,080	1.0		1.0	
	100–250	15.6	[13.6; 17.9]	217	1,391	1.8*	[1.3; 2.3]	1.8*	[1.4; 2.4]
	<100	22.0	[16.2; 29.2]	40	182	2.7*	[1.7; 4.1]	2.8*	[1.8; 4.4]
**Age groups (years)**									
	0.5–5	6.7	[4.7; 9.4]	29	433	1.0		1.0°	
	6–10	8.9	[6.5; 12.1]	36	404	1.4	[0.8; 2.2]		
	11–19	13.9	[10.7; 16.7]	68	491	2.2*	[1.4; 3.5]	2.1*^∞^	[1.6; 2.8]
	20–39	15.8	[13.4; 18.4]	140	889	2.6*	[1.7; 3.9]		
	> = 40	20.0	[16.5; 24.0]	87	436	3.5*	[2.2; 5.4]	3.1*	[2.2; 4.4]
**Education**									
	None	8.1	[6; 10.8]	39	483	1.0		-	
	Primary	13.3	[11.3; 15.7]	140	1,199	1.8*	[1.2; 2.5]	-	
	Secondary	16.3	[14; 19]	153	829	2.2*	[1.5; 3.2]	-	
	Superior	15.1	[10.7; 20.9]	27	141	2.0*	[1.2; 3.4]	-	
**Income amount**									
	None	12.6	[11.1; 14.3]	261	2,067	1.0		-	
	< 650 PEN	17.9	[14.7; 21.7]	82	458	1.5*	[1.2; 2]	-	
	> = 650 PEN	14.5	[8.4; 23.8]	12	83	1.3	[0.6; 2.2]	-	
**Occupation as farmer** **(previous 12 months)**									
	No	13.3	[11.9; 14.8]	344	2,590	1.0		-	
	Yes	25.4	[16.3; 37.3]	16	63	2.2*	[1.3; 3.9]	-	
**Occupation as moto-taxi driver (previous 12 months)**									
	Never/Sometimes	13.4	[12.1; 14.9]	347	2,584	1.0		-	
	Always	18.8	[11.2; 30.0]	13	69	1.5	[0.8; 2.8]	-	
**Bed net use**									
	No	13.0	[11.1; 15.2]	162	1,244	1.0		-	
	Yes	14.1	[12.2; 16.2]	196	1,395	1.1	[0.9; 1.4]	-	
**Confirmed *P. vivax *malaria (previous 12 months)+**									
	No	10.5	[9.3; 12]	247	2,346	1.0		-	
	Yes (1 episode)	33.3	[27.9; 39.3]	91	273	4.2*	[3.2; 5.7]	-	
	Yes (> = 2episodes)	64.7	[46.7; 79.3]	22	34	15.6*	[7.3; 33]	-	

* p < 0.05. + Not considered in the multivariate analysis.

° Baseline category = 0.5–10 years.

^∞^ Include ages between 11 and 39 years.

**≈** A significant interaction (OR = 5.7; 95CI [1.4; 22.7], p = 0.014) was found between age >10 years and distance <100 m.

Significant risk factors for *P. falciparum* exposure identified in the univariate analysis were the following: settlement, age, education, prior work as farmer or moto-taxi driver, and bed net use (Table 5). The multivariate-adjusted analysis showed that only settlement, age and previous work as moto-taxi driver remained independently associated with exposure to *P. falciparum* (no interaction was found). Individuals living in JCM (AOR: 1.5, 95% CI [1.02–2.1]) and in NP (AOR: 2.2, 95% CI [1.6–3.2]) were more likely to be exposed to *P. falciparum* compared to those living in PAV. In addition, individuals older than five years had increasingly higher odds (test for trend p < 0.001) of *P. falciparum* seropositivity compared to younger children, and those having worked as moto-taxi driver in the previous year were two times more exposed to *P. falciparum* (AOR: 2.1, 95% CI [1.1–3.8]) compared to those who did not.

**Table 5:** **Multivariate adjusted analysis for the risk of exposure to *Plasmodium falciparum *using survey logistic regression**

**Risk factors**		**Positive Pf GLURP-19**				**Unadjusted**		**Adjusted**	
		**%**	**95% CI**	**n**	**N**	**OR**	**95% CI**	**OR**	**95% CI**
**Settlement**									
	PAV	7.4	[5.9; 9.3]	83	1,123	1.0		1.0	
	JCM	9.6	[7.6; 12.2]	76	789	1.3	[0.9; 1.9]	1.5*	[1.02; 2.1]
	NP	13.6	[11; 16.5]	100	738	2.0*	[1.4; 2.8]	2.2*	[1.6; 3.2]
**House distance to water drain (m)**									
	>250	10.6	[8.7; 12.8]	114	1,080	1.0		-	
	100–250	8.7	[7.3; 10.4]	121	1,388	0.8	[0.6; 1.1]	-	
	<100	13.2	[8.7; 19.5]	24	182	1.3	[0.8; 2.2]	-	
**Age groups (years)**									
	0.5–5	3.7	[2.9; 7.4]	16	432	1.0		1.0	
	6–10	7.4	[3.4; 8.0]	30	404	2.1*	[1.1; 3.9]	2.1*	[1.1; 3.8]
	11–19	12.5	[9.7; 15.9]	61	490	3.7*	[2.1; 6.5]	3.2*^∞^	[1.9; 5.3]
	20–39	10.3	[8.4; 12.5]	91	888	3.0*	[1.8; 5.0]		
	> = 40	14.0	[11.0; 17.6]	61	436	4.2*	[2.4; 7.4]	4.7*	[2.6; 8.3]
**Education**									
	None	6.0	[4.2; 8.5]	29	483	1.0		-	
	Primary	10.0	[8.3; 12]	105	1,051	1.7*	[1.1; 2.7]	-	
	Secondary	11.1	[9.2; 13.4]	104	937	2.0*	[1.3; 3]	-	
	Superior	11.8	[7.8; 17.6]	21	178	2.1*	[1.2; 3.8]	-	
**Income amount**									
	None	9.2	[8; 10.6]	190	2,064	1.0		-	
	< 650 PEN	11.8	[9.1; 15.1]	54	458	1.3	[0.96; 1.8]	-	
	> = 650 PEN	14.5	[8.4; 23.8]	12	83	1.7	[0.9; 3.1]	-	
**Occupation as farmer (previous 12 months)**									
	No	9.6	[8.4; 10.9]	248	2,587	1.0		-	
	Yes	17.5	[9.9; 29]	11	63	2.0*	[1.03; 3.9]	-	
**Occupation as moto-taxi driver (previous 12 months)**									
	No	9.5	[8.3; 10.8]	245	2,581	1.0		1.0	
	Yes	20.3	[12.4; 31.4]	14	69	2.4*	[1.3; 4.4]	2.1*	[1.1; 3.8]
**Bed net use**									
	Never/Sometimes	11.3	[9.5; 13.4]	141	1,244	1.0		-	
	Always	8.5	[7.1; 10.2]	118	1,392	0.7*	[0.6; 0.96]	-	

* p < 0.05.

^∞^ Included all ages between 11 and 39 years.

**Figure 2:**
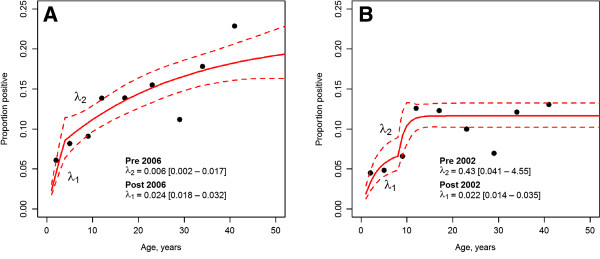
**Malaria seroprevalence curves. A**: Seroprevalence curve for *Plasmodium vivax*. **B**: Seroprevalence curve for *Plasmodium falciparum*. Points represent observed data points, whilst the blue lines represent the maximum likelihood model and 95% confidence intervals. Estimated force of infection (λ) is plotted on the graphs. Two forces infection are plotted by likelihood ratio tests indicating change at a certain point in calendar time.

Age seroprevalence plots for PvMSP1_19_ and PfGLURP are shown in Figure 2 (A and B), and confirm previous results: increasing *P. vivax* and *P. falciparum* seroprevalence with age. Likelihood ratio (LR) tests supported models with two SCR (forces of infection) rather than one SCR for both *P. vivax* (p = 0.028) and *P. falciparum* (p = 0.046), indicating significant changes over time in the MTI for both species. For PvMSP1_19_, best estimates indicated a significant four-fold increase in the SCR approximately four years prior to the survey (year 2006): post-2006 **λ**_**1**_ = 0.024 (95% CI [0.018–0.032]) and pre-2006 **λ**_**2**_ = 0.006 (95% CI [0.002–0.017]). For PfGLURP, best estimates indicated a 19–fold drop in the SCR approximately eight years prior to the survey (year 2002): post-2002 **λ**_**1**_ = 0.022 (95% CI [0.014–0.033]), and pre-2002 **λ**_**2**_ = 0.431 (95% CI [0.041–4.54]).

**Figure 3:**
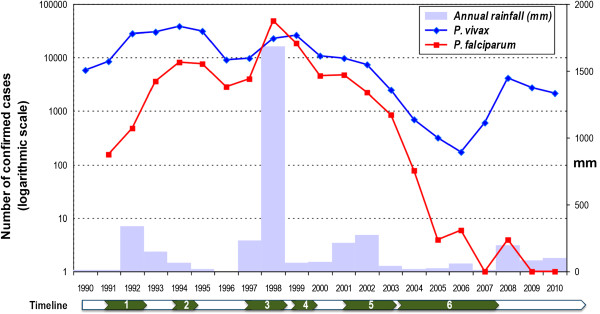
**Evolution of the annual malaria incidence, and rainfall, in Piura department: 1990–2010.** Information on annual malaria cases, registered by Regional Health Direction of Piura, was obtained from the National Institute of Statistics and Informatics [27]. Numbers in the timeline point out important events that influenced malaria incidence: 1) moderate ENSO phenomenon (1991–1992), 2) first reports of chloroquine (CQ) failure for *Plasmodium falciparum* (since 1994), 3) very strong ENSO phenomenon (1997–1998), 4) confirmed CQ resistance for *Plasmodium falciparum* (1999), 5) ACT implementation (2001–2003), 6) intense droughts (2004–2007).

The retrospective analysis of 20-year annual malaria incidence and rainfall from 1990 to 2010 in Piura department showed that after the El Niño-Southern Oscillation (ENSO) phenomenon in 1997–1998 (Figure 3), when nearly 50,000 *P. falciparum* cases were recorded (1998), *P. falciparum* incidence dropped and stabilized around 4,000–5,000 cases annually in 2000–2001. This was followed by a steady decrease, and since 2006, autochthonous *P. falciparum* reported cases have become scarce (six and three reported cases in 2006 and 2008, respectively; and no cases in 2007, 2009 and 2010). Similarly to *P. falciparum*, *P. vivax* incidence decreased gradually after the ENSO (around 23,000 cases in 1998) reaching very low levels during the four-year period of intense droughts, with only 700, 315, 168, and 606 reported cases, respectively from 2004 to 2007. However, since then, *P. vivax* cases have increased with 4,185, 2,735 and 2,153 cases in 2008, 2009 and 2010, respectively. During the same period, annual rainfall in Piura dramatically dropped from 1998 (1,687 mm) to minimum values (between 14.3 and 59.4 mm) during the drought period, but subsequently increased since 2008 (193.5 mm in 2008).

## Discussion

The combination of molecular and serological tools, in addition to standard microscopy, allowed for an in-depth characterization of the current malaria transmission pattern as well as recent changes in species-specific MTI in this peri-urban area of the Peruvian northern coast. Most of the malaria infections were submicroscopic (70%), as well as asymptomatic (89%) despite the low level of transmission. Though *P. vivax* was predominant, species-specific seroprevalence showed that exposure to *P. falciparum* still occurs in the study area. Interestingly, over the past ten years, both species experienced significant but opposite changes in MTI: while *P. falciparum* MTI dropped after 2002, the one of *P. vivax* significantly increased after 2006.

Despite having been conducted at the end of rainy season, when the health information system usually records the highest malaria incidence [7], few malaria-infected individuals were identified and most of them had not had malaria-related symptoms within the two days prior to the survey. These results are in line with findings from other areas in the northern coast [39] and the Peruvian Amazon region [40,41] and support the hypothesis that this asymptomatic and submicroscopic reservoir of infections contributes to malaria transmission [42].

In areas of low malaria transmission with seasonal patterns affected by climatic conditions, such as the Peruvian northern coast region, parasitological indicators determined by microscopy or PCR can be insensitive. In these areas, serological measures can be used as a proxy for malaria transmission intensity [20,23,25,36]. Indeed, seroprevalence reflects the cumulative exposure to malaria and, since antibodies persist longer in the blood than parasites, it is more sensitive and less affected by seasonality or unstable transmission [22,23,43]. Though it may be limited in detecting discrete seasonal variations in transmission, it is a good indicator of long-term transmission potential [22,24].

Even though *P. falciparum* reported cases in Piura department have been extremely rare in the past five years, *P. vivax* and *P. falciparum* seroprevalence indicated ongoing exposure to both species. The low PfGLURP seropositivity in young children and the only one *P. falciparum* case detected by PCR suggest that a residual *P. falciparum* transmission is still present in the study area. This residual transmission may be due to asymptomatic *P. falciparum* infections that remain undetected, unless a population-wide survey (such as the present study) is carried out. However, the implementation of mass screening as a public health strategy in low transmission settings remains a matter of debate [18,40,41], as the cost-effectiveness of such an intervention remains difficult to establish. The role of asymptomatic infections in maintaining malaria transmission as well as the impact of mass screening and treatment should be further evaluated through modelling techniques [44].

One might argue that an alternative explanation for the observed *P. falciparum* seropositivity could be the presence of antibodies cross-reacting with *P. vivax* or other infectious agents’ antigens. Limited information is available about the cross-reactivity between *P. falciparum* and *P. vivax* antigens [35,45], and this has not been reported for PfGLURP. In addition, the lack of association between confirmed *P. vivax* infections by PCR and PfGLURP seropositivity, and the absence of correlation between antibody responses to the two species suggest that cross-reactivity is probably minimal. A few studies have suggested the cross-reactive potential of PfGLURP with *Schistosoma haematobium*[46], which is unlikely in the study area since the latter has not been reported in Piura.

Serologic markers can also be used to determine risk factors for malaria. This is particularly important in low endemicity areas as the one reported here, where the low malaria prevalence jeopardizes the risk factor analysis. In the study area, age was the only independent risk factor that was associated to both species. The age-dependent PvMSP119 and PfGLURP seropositivity indicates cumulative *P. vivax* and *P. falciparum* exposure with increasing age. As antibody responses will persist for prolonged periods, the rate of acquisition in young age groups can be used to determine current malaria transmission intensity [22-25] as well as to identify recent changes in MTI.

The higher seroprevalence in individuals with several *P. vivax* malaria episodes also confirmed that PvMSP119 antibody detection measures cumulative exposure. However, the history of *P. vivax* malaria in the past year was not included in the multivariate risk factor analysis for *P. vivax* exposure, since it is not a risk factor for *P. vivax* infections *per se* but rather a consequence of previous exposure to a given risk factor. *Plasmodium vivax* infections are on the pathway between risk factors and the presence of *P. vivax* antibodies.

Differences in the risk factors for *P. vivax* and *P. falciparum* seropositivity reflect the heterogeneity of malaria transmission intensity between species. Age and distance to the water drain were the only two independent risk factors significantly associated with *P. vivax* exposure. As mentioned above, the effect of age on seroprevalence usually reflects the cumulative exposure to malaria infections occurring with age rather than a higher risk of exposure in adults as compared to children. However, the significant interaction found between age and house distance to the drain, beside the cumulative effect of age on seroprevalence, indicates a significant lower exposure in children aged < ten years. Indeed, the effect of distance (<100 m *versus* ≥100 m) on *P. vivax* seropositivity was only significant in adults, indicating that children living close to the drain were not exposed any more to infectious bites compared to children living farther. Water accumulation in the drain provides breeding habitats for *Anopheles* malaria vectors after rains [30,39], resulting in a higher risk of exposure to infectious vectors for people living close to the drain. This is also supported by the results of univariate analysis for the risk of *P. vivax* infection. However, since adults are more likely to have outdoor evening activities, this could explain why children below ten, were no more at risk of malaria while living close to the drain.

Besides age, settlement and prior work as moto-taxi driver in the previous year, there were two additional independent risk factors for *P. falciparum* seropositivity. Historically, the three study settlements were important foci of *P. falciparum* transmission during the epidemic of 1998 and the early 2000s [47,48]. Therefore, higher seroprevalence in JCM and NP, compared to PAV again suggest differential reduction rates in MTI between villages (heterogeneity). Occupational activities outside settlements (as moto-taxi driver) that were associated with increased *P. falciparum* exposure support the hypothesis that transmission occurs not only in the immediate house environment as previously described by Guthmann *et al*. [29], but outside the community as well. Moto-taxi drivers in the study area usually work in evening times, when exposure to *An. albimanus* bites increases. Occupational risk for malaria has also been found in the Peruvian Amazon Region, where malaria morbidity has been clearly associated with forest-related activities such as farming, land clearing and wood extraction [49,50].

The catalytic model used to generate PfGLURP seroprevalence curves suggested that a significant decline in *P. falciparum* transmission occurred in the study area approximately eight years before the current study (around 2002). This supports the idea that the wide scale implementation of ACT since 2001 has significantly contributed to the reduction of *P. falciparum* MTI. Indeed, following the 1998 epidemic and the subsequent intensified control interventions implemented [9], *P. falciparum* incidence returned to the pre-epidemic levels (1997) by 2000–2001 (around 5,000 cases), and since 2002 it steadily decreased to reach almost undetected levels from 2007 onwards (Figure 3). In addition, the absence of *P. falciparum* surge after the drought period in 2007, as observed with *P. vivax,* advocates for the additional significant effect of the ACT deployment on *P falciparum* MTI in the northern coast region [8].

Since the end of the drought period and the resuming of the rains, *P. vivax* incidence substantially increased [7] (Figure 3) which is concordant with the fitted results from the model, i.e., significant increase in *P. vivax* MTI after 2006. *Plasmodium vivax* transmission in this area seems to be more sensitive than *P. falciparum* to changes in climatological and environmental conditions, especially since the implementation of ACT. The recent changes in species specific MTIs are also in line with the results of the multivariate risk factor analysis. While children under-five were significantly at lower risk of *P. falciparum* exposure compared to older children age 6-10, this was not the case for the *P. vivax* exposure where the under five were at similar risk of exposure compared to the 6-10 years old.

The number of antigens used in the two serological tests could be a limitation of the study. Due to the degree of sequence variability, it is recommended to use multiple antigens for each species in order to optimize the sensitivity of the antibody detection and to improve the identification of changes in malaria transmission over time in low transmission settings [20,38]. However, the observation that the antigens used in the study are relatively conserved [51] combined with results from recent studies performed in Cambodia, Vietnam and Peru suggests that PfGLURP and PvMSP1_19_ are highly suitable for the detection of antibodies against *P. falciparum* and *P. vivax*, respectively [33,52]*.* Multiplexing the serological tests as well as standardizing the antigens used is of crucial importance for monitoring elimination efforts and comparing trends across countries and regions.

## Conclusions

Monitoring MTI in areas of low endemicity and seasonal patterns, such as the north-western coast of Peru, poses a major challenge to national malaria control programmes. In this setting, molecular tools confirmed the current malaria transmission pattern characterized by low parasite rates, mainly due to *P. vivax* with a majority of asymptomatic and sub-microscopic infections. Additionally, serological markers reflected exposure to malaria and allowed for species-specific risk factor analysis, as well as showing recent changes in respective MTIs. In conclusion, the combination of both serological and molecular tools improved detection sensitivity, and provided new insights into recent changes in transmission intensity for both species. This invaluable information can help decision-makers to adjust malaria control strategies in order to maintain current achievements, avoid outbreaks, and possibly move towards the elimination of malaria.

## Competing interests

The authors declare that they have no competing interests.

## Authors’ contributions

AR contributed to the study design, analysed the data and wrote the paper; ALL and VS contributed to the study design and paper review; NS and JC contributed to the data analysis and paper review; JCM contributed to the fieldwork supervision and processed the blood samples with ELISA and PCR; EP and OJP carried out and coordinated the fieldwork; DG contributed to the supervision of the sample processing and paper review; MOP, ISS and MT contributed antigen to the project and reviewed the article; UDA contributed to the study design and reviewed the manuscript; AE contributed to the study design, the data analysis and reviewed the paper. All authors read and approved the final manuscript.
